# A Transplantable Phosphorylation Probe for Direct Assessment of G Protein-Coupled Receptor Activation

**DOI:** 10.1371/journal.pone.0039458

**Published:** 2012-06-26

**Authors:** Andrea Kliewer, Anika Mann, Aline Petrich, Florian Pöll, Stefan Schulz

**Affiliations:** Department of Pharmacology and Toxicology, Jena University Hospital - Friedrich Schiller University Jena, Jena, Germany; University of Cordoba, Spain

## Abstract

The newly developed multireceptor somatostatin analogs pasireotide (SOM230), octreotide and somatoprim (DG3173) have primarily been characterized according to their binding profiles. However, their ability to activate individual somatostatin receptor subtypes (sst) has not been directly assessed so far. Here, we transplanted the carboxyl-terminal phosphorylation motif of the sst_2_ receptor to other somatostatin receptors and assessed receptor activation using a set of three phosphosite-specific antibodies. Our comparative analysis revealed unexpected efficacy profiles for pasireotide, octreotide and somatoprim. Pasireotide was able to activate sst_3_ and sst_5_ receptors but was only a partial agonist at the sst_2_ receptor. Octreotide exhibited potent agonistic properties at the sst_2_ receptor but produced very little sst_5_ receptor activation. Like octreotide, somatoprim was a full agonist at the sst_2_ receptor. Unlike octreotide, somatoprim was also a potent agonist at the sst_5_ receptor. Together, we propose the application of a phosphorylation probe for direct assessment of G protein-coupled receptor activation and demonstrate its utility in the pharmacological characterization of novel somatostatin analogs.

## Introduction

The development of novel multireceptor somatostatin analogs has primarily focused on the discovery of compounds with nanomolar binding affinities to more than one of the five somatostatin receptors (sst_1_–sst_5_). It is not clear, however, whether these compounds exhibit full or partial agonistic properties at individual somatostatin receptor subtypes. This lack of knowledge is due to the limited availability of methods allowing a direct assessment of G protein-coupled receptor (GPCR) activation.

In clinical practice, octreotide and lanreotide are used as first choice medical treatment of neuroendocrine tumors such as GH-secreting adenomas and carcinoids [1], [2]. Octreotide and lanreotide bind with high sub-nanomolar affinity to sst_2_ only, have moderate affinity to sst_3_ and sst_5_ and show very low or absent binding to sst_1_ and sst_4_. Recently, the novel multireceptor somatostatin analog, pasireotide (SOM230), has been synthesized [3]. Pasireotide is a cyclohexapeptide, which binds with high affinity to all somatostatin receptors except to sst_4_ [4]. In contrast to octreotide, pasireotide exhibits particular high sub-nanomolar affinity to sst_5_ [5]. Pasireotide is currently under clinical evaluation for treatment of acromegaly, Cushing’s disease and octreotide-resistant carcinoid tumors [6], [7], [8]. In addition to pasireotide, the novel pan-somatostatin analog somatoprim (DG3173) is currently under clinical and preclinical evaluation. Somatoprim exhibits a unique binding profile in that binds with high affinity to sst_2_, sst_4_ and sst_5_ but not to sst_1_ or sst_3_.

We have recently uncovered agonist-selective and species-specific patterns of sst_2A_ receptor phosphorylation and trafficking [9]. Whereas octreotide, in a manner similar to that observed with somatostatin, stimulates the phosphorylation of a number of carboxyl-terminal phosphate acceptor sites in both rat and human sst_2_ receptors, pasireotide fails to promote any detectable phosphorylation or internalization of the rat sst_2A_ receptor. In contrast, pasireotide is able to trigger a partial internalization of the human sst_2_ receptor. At present it is unclear whether the agonist-selective regulation of the sst_2_ receptor observed for pasireotide is a general property of all pan-somatostatin analogs, and whether such functional selectivity may exist for other clinically-relevant somatostatin receptors including sst_5_ and sst_3_.

In the present study, we addressed this problem by using the carboxyl-terminal tail of the sst_2_ receptor as transplantable phosphorylation probe to directly sense the activation of other somatostatin receptors. This approach was possible due to our recent success in generating a set of three phosphosite-specific antibodies for the sst_2_ receptor which allowed us to determine distinct patterns of phosphorylation induced by different agonists. Our assay utilizes the unique ability of G protein-coupled receptor kinases (GRKs) to detect only active conformations of GPCRs. Different phosphorylation patterns may hence reflect distinct receptor conformations.

## Materials and Methods

### Reagents and Antibodies

Pasireotide and octreotide were provided by Dr. Herbert Schmid (Novartis, Basel, Switzerland). Somatoprim was provided by Dr. Ursula Hoffmann (DeveloGen, Göttingen, Germany). Somatostatin (SS-14) was obtained from Bachem (Weil am Rhein, Germany). The phosphorylation-independent rabbit monoclonal anti-sst_2_ {UMB-1}, anti-sst_3_ {UMB-5} or anti-sst_5_ {UMB-4} antibodies were obtained from Epitomics (Burlingame, CA). The rabbit polyclonal phosphosite-specific sst_2_ antibodies anti-pT353/pT354 {0521}, anti-pT356/pT359 {0522}, and anti-pS341/pS343 {3155} were generated and extensively characterized previously [9], [10].

**Figure 1:**
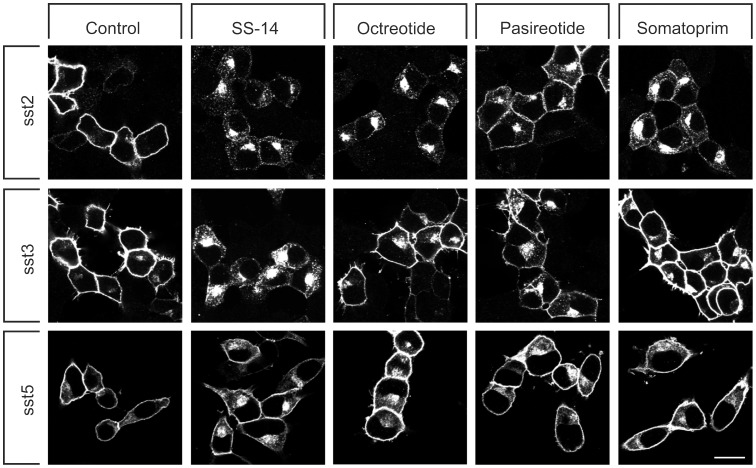
Agonist-selective internalization of human somatostatin receptors. HEK293 cells stably expressing sst_2_, sst_3_ or sst_5_ receptors were treated with either 1 µM SS-14, octreotide, pasireotide or somatoprim for 0 or 30 min. Cells were fixed, immunoflurescently stained with anti-sst_2_ {UMB-1}, anti-sst_3_ {UMB-5} or anti-sst_5_ {UMB-4} antibodies, and examined by confocal microscopy. Shown are representative images from one of three independent experiments performed in duplicate. *Scale bar*, 20 µm.

**Figure 2:**
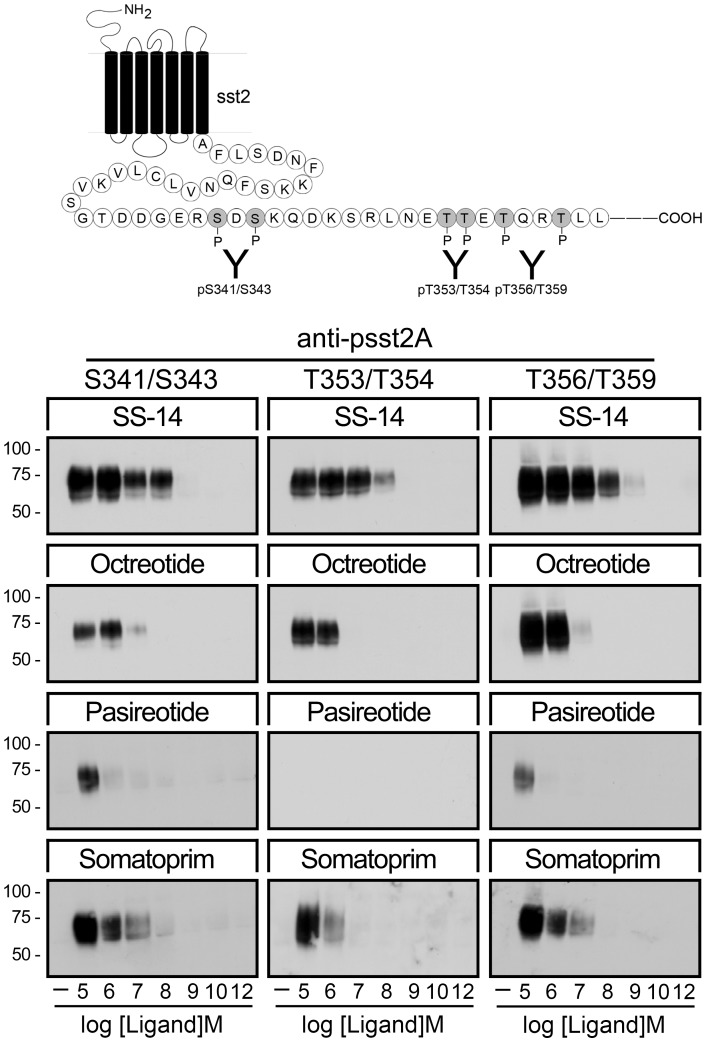
Agonist-selective phosphorylation of the human sst_2_ receptor. (*Top*) Schematic representation of the human sst_2_ receptor indicating the phosphate acceptor sites S341/343, T353/354 and T356/359 within its carboxyl-terminal tail. (*Bottom*) HEK293 cells stably expressing the sst_2_ receptor were either not exposed or exposed for 5 min to SS-14, octreotide, pasireotide or somatoprim in concentrations ranging from 10^−12^ to 10^−5^ M. The levels of phosphorylated sst_2_ receptors were then determined using the phosphosite-specific antibodies anti-pS341/pS343 {3157}, anti-pT353/pT354 {0521} and anti-pT356/pT359 {0522}. Western blots shown are representative of three to five independent experiments for each condition. The positions of the molecular mass markers are indicated on the left (in kDa).

### Generation of Mutant Somatostatin Receptors

A chimera of the human sst_5_ receptor with the carboxyl-terminal tail of the human sst_2_ receptor (hsst5-sst2CT) was generated by DNA synthesis by imaGenes (Berlin, Germany). A chimera of the rat sst_3_ receptor with the carboxyl-terminal tail of the rat sst_2_ receptor (rsst3-sst2ACT) was generated by exchange of the entire carboxyl-terminal tail using the FLS Motive present in the DNA sequence of both receptors in the seventh transmembrane domain. The fragments were cloned into pcDNA3.1(+) using HindIII and XbaI cloning sites.

**Figure 3:**
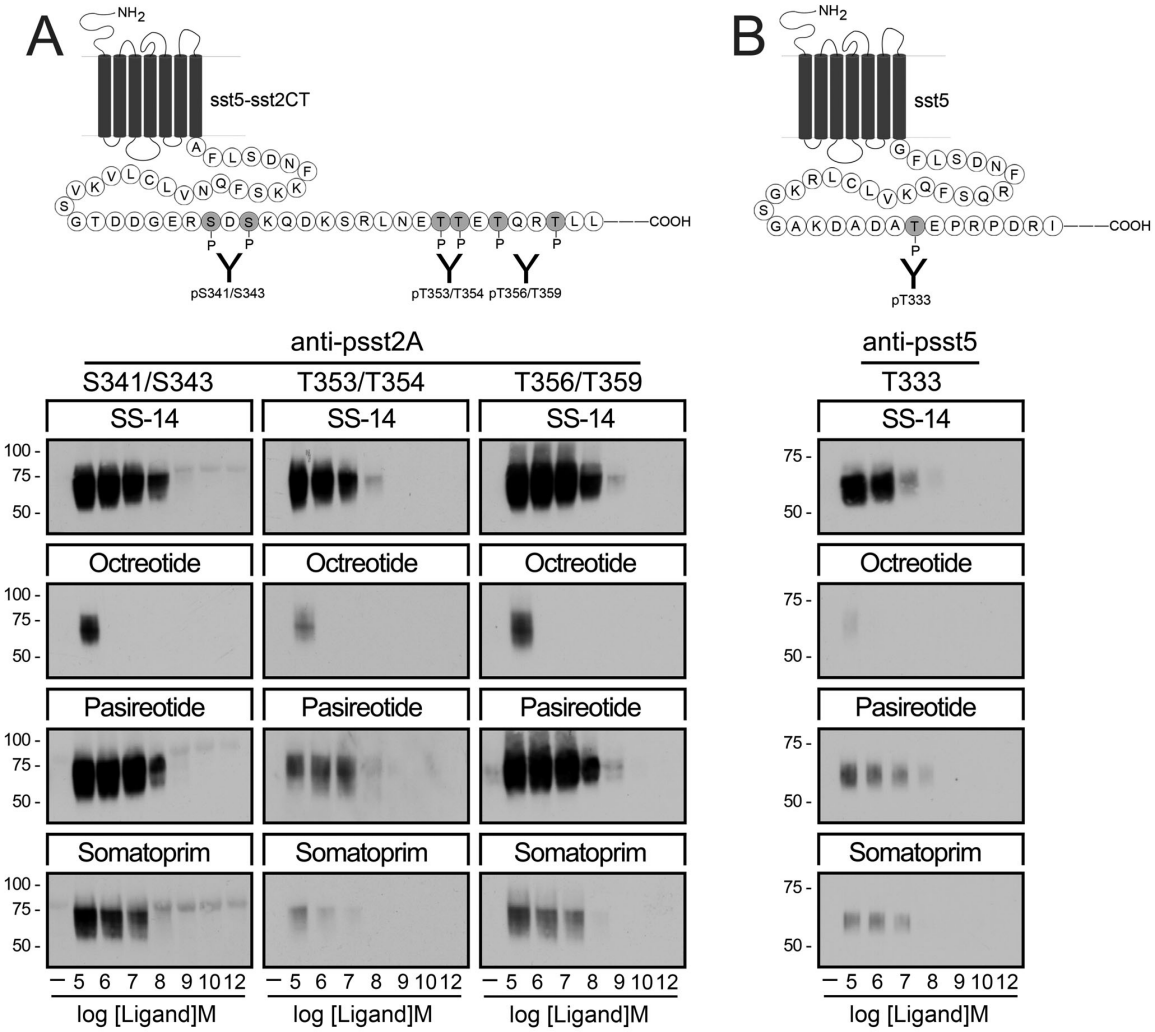
Agonist-selective phosphorylation of the human sst_5_ and sst_5_-sst_2_CT chimera. (*A*,*Top panel*) Schematic representation of the sst_5_-sst_2_CT receptor indicating the phosphate acceptor sites S341/343, T353/354 and T356/359 within the carboxyl-terminal tail. (*A*, *Bottom*) HEK293 cells stably expressing the sst_5_-sst_2A_CT receptor were either not exposed or exposed for 5 min to SS-14, octreotide, pasireotide or somatoprim in concentrations ranging from 10^−12^ to 10^−5^ M. The levels of phosphorylated sst_5_-sst_2A_CT receptors were then determined using the phosphosite-specific antibodies anti-pS341/pS343 {3157}, anti-pT353/pT354 {0521} and anti-pT356/pT359 {0522}. (*B*,*Top panel*) Schematic representation of the human sst_5_ receptor indicating the phosphate acceptor site T333 within its carboxyl-terminal tail. (*B*, *Bottom*) HEK293 cells stably expressing the human sst_5_ receptors were either not exposed or exposed for 5 min to SS-14, octreotide, pasireotide or somatoprim in concentrations ranging from 10^−12^ to 10^−5^ M. The levels of phosphorylated sst_5_ receptors were then determined using the phosphosite-specific antibodies anti-pT333 {3567}. Western blots shown are representative of three to five independent experiments for each condition. The positions of the molecular mass markers are indicated on the left (in kDa).

**Table 1:** Ligand binding properties of sst_5_-sst_2_CT receptors.

Ligand	Ligand binding affinity IC_50_ (nM)		
	human sst_2_	human sst_5_	human sst_5_-2CT
SS-14	5.7±1.1	15.5±2.6	30.3±5.0
Octreotide	1.4±0.3	28.9±4.2	38.8±11.7
Pasireotide	21.3±5.7	3.6±1.5	10.8±5.3
Somatoprim	4.7±0.6	5.6±2.2	5.4±1.6

Ligand binding assays were carried out as described under “Materials and Methods”. The half-maximal inhibitory concentrations (IC_50_) were analyzed by nonlinear regression curve fitting using the computer program GraphPad Prism. Data are presented as the mean of three independent experiments performed in triplicate.

### Cell Culture and Transfection

Human embryonic kidney HEK293 cells were obtained from the German Resource Centre for Biological Material (DSMZ, Braunschweig, Germany). HEK293 cells were grown in Dulbecco’s modified Eagle’s medium supplemented with 10% fetal calf serum in a humidified atmosphere containing 10% CO_2_. Cells were transfected with plasmids encoding for wild-type or mutant somatostatin receptors using jetPEI™ according to the instructions of the manufacturer (Invitrogen, Carlsbad, CA). Stable transfectants were selected in the presence of 400 µg/ml G418. HEK293 cells stably expressing somatostatin receptors were characterized using radioligand-binding assays, Western blot analysis, and immunocytochemistry as described previously. The level of somatostatin receptor expression was ∼900 fmol/mg membrane protein. All chimeras and mutants tested were present at the cell surface and expressed similar amounts of receptor protein. The IC_50_ values of SS-14, octreotide and pasireotide for ligand binding affinities are given in Table S1.

**Figure 4:**
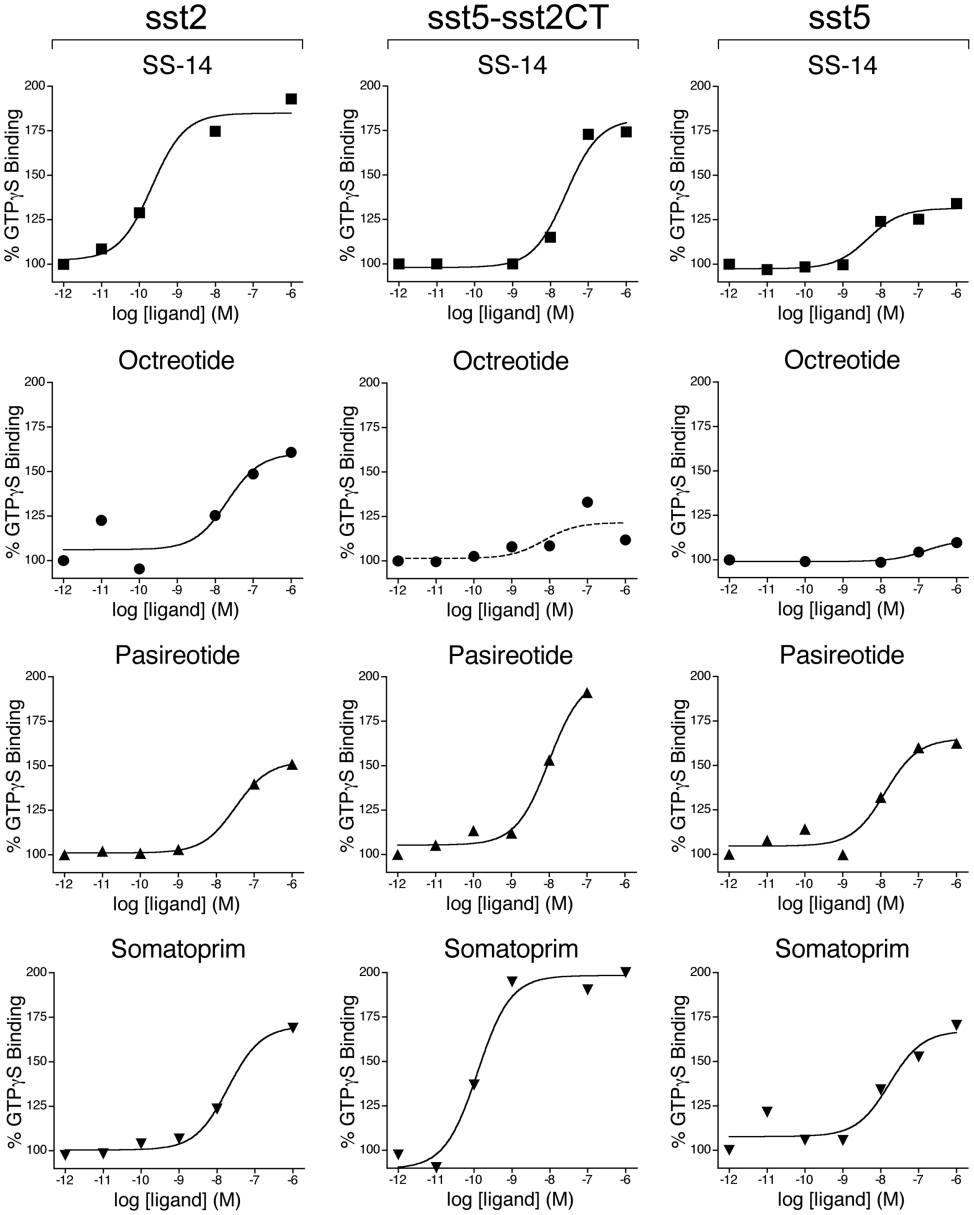
Agonist-stimulated ^35^S-GTPγS binding. Stimulation of [^35^S]GTPγS binding by SS-14, Octreotide, Pasireotide and Somatoprim in the concentration range of 10^−12^ to 10^−6^ M. Membranes wer prepared from HEK293 cells stably expressing either the human sst_2_, sst_5_ and sst_5_-sst_2A_CT or the rat sst_3_-sst_2A_CT receptor. Values represent means of triplicate determinations. SE values were smaller than 15%. Three replicate experiments gave similar results.

**Figure 5:**
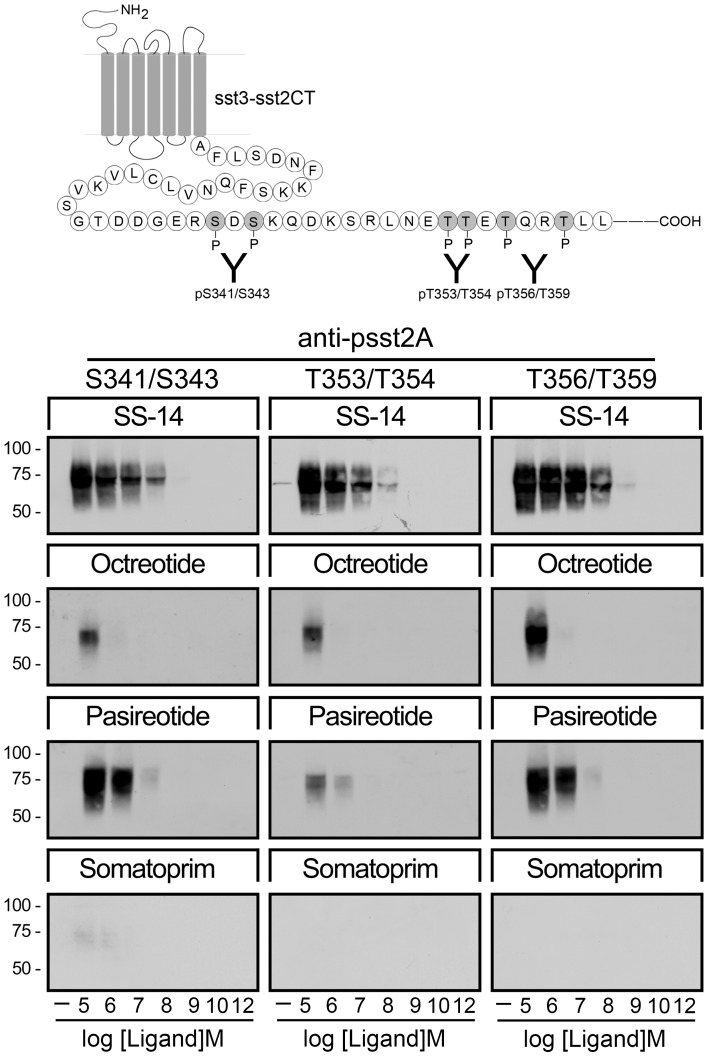
Agonist-selective phosphorylation of the sst_3_-sst_2_CT chimera. (*Top*) Schematic representation of the rat sst_3_-sst_2_CT chimera indicating the phosphate acceptor sites S341/343, T353/354 and T356/359 within the carboxyl-terminal tail. (*Bottom*) HEK293 cells stably expressing the rat sst_3_-sst_2A_CT receptor were either not exposed or exposed to concentrations of 10^−12^ to 10^−5^ M SS-14, octreotide, pasireotide or somatoprim for 5 min. The levels of phosphorylated rsst_3_-sst_2A_CT receptors were then determined using anti-pS341/pS343 {3157}, anti-pT353/pT354 {0521} or anti-pT356/pT359 {0522}. Western blots shown are representative of three to five independent experiments for each condition. The positions of the molecular mass markers are indicated on the left (in kDa).

### Immunocytochemistry

Cells were grown on poly-L-lysine-coated coverslips overnight. After the appropriate treatment with SS-14, octreotide, pasireotide or somatoprim, cells were fixed with 4% paraformaldehyde and 0.2% picric acid in phosphate buffer (pH 6.9) for 30 min at room temperature and washed several times. Specimens were permeabilized and then incubated with anti-sst_2_ {UMB-1}, anti-sst_3_ {UMB-5} or anti-sst_5_ {UMB-4} antibodies followed by Alexa488-conjugated secondary antibodies (Amersham, Braunschweig, Germany). Specimens were mounted and examined using a Zeiss LSM510 META laser scanning confocal microscope [11].

### Western Blot Analysis

Stably transfected HEK293 cells were plated onto poly-L-lysine-coated 60-mm dishes and grown to 80% confluence. After the appropriate treatment with SS-14, octreotide, pasireotide or somatoprim, cells were lysed in detergent buffer (50 mM Tris-HCl, pH 7.4, 150 mM NaCl, 5 mM EDTA, 10 mM NaF, 10 mM disodium pyrophosphate, 1% Nonidet P-40, 0.5% sodium deoxycholate, 0.1% SDS, 0.2 mM phenylmethylsulfonyl fluoride, 10 µg/ml leupeptin, 1 µg/ml pepstatin A, 1 µg/ml aprotinin, and 10 µg/ml bacitracin). Glycosylated proteins were partially enriched using wheat germ lectin-agarose beads as described [12], [13], [14]. Proteins were eluted from the beads using SDS-sample buffer for 20 min at 60°C and then resolved on 10% SDS-polyacrylamide gels. After electroblotting, membranes were incubated with the phosphosite-specific antibodies anti-pS341/pS343 {3157}, anti-pT353/pT354 {0521} or anti-pT356/pT359 {0522} at a concentration of 0.1 µg/ml followed by detection using enhanced chemiluminescence (Amersham). Blots were subsequently stripped and reprobed with anti-sst_2_ {UMB-1} to confirm equal loading of the gels.

### Radioligand Binding Assay

Competition binding assays were performed on membrane preparations from stable transfected cells as described above. Cells were harvested into PBS and stored at –80°C. After thawing, cells were centrifuged at 20,000×*g* for 10 min at 4°C and then homogenized in lysis buffer (50 mM Tris-HCl, 3 mM EGTA, 5 mM EDTA, pH 7.4). Cell membranes were pelleted by centrifugation at 50,000×*g* for 15 min at 4°C, washed twice with washing buffer (50 mM Tris-HCl, ph 7.4), and resuspended in binding buffer (10 mM HEPES, 5 mM MgCl_2_, 5 µg/ml bacitracin, pH 7.5). For competition binding assay, aliquots of the membrane preparations containing 30 µg of protein were incubated with 0.05 nM [^125^J-Tyr11]-SS-14 (specific activity: 74 TBq/mmol, PerkinElmer, USA) in the present or absence of either SS-14, octreotide, pasireotide or somatoprim in concentrations ranging from 10^−12^ to 10^−6^ M. The experiment for each concentration was performed in triplicate. Assays were performed in 96-well polypropylene plates in a final volume of 200 µl for 45 min at room temperature. Specific binding was calculated by subtracting non-specific binding – defined as that seen in the presence of 1 µM SS-14, octreotide, pasireotide or somatoprim – from total binding obtained with radioligand alone. The incubation was terminated by addition of ice-cold buffer and rapid vacuum filtration through glass fiber filters presoaked in 0.3% polyethyleneimine using an Inotech cell harvester (Dittikon, Switzerland). Filters were rinsed twice with washing buffer and air-dried. Bound radioactivity was determined using a γ-counter (COBRAII, Packard, USA). Data from ligand binding and IC_50_ were analyzed by curve fitting using GraphPad Prism 4.0 software [15].

### GTPγS Binding Assays

Cells were harvested and lysed as described above except that a lysis buffer containing 50 mM Tris, 10 mM EDTA and 1 mM EGTA (pH 7.4) was used. The resulting pellet was resuspended in assay buffer (20 mM HEPES, 100 mM NaCl, 10 mM MgCl_2_, pH 7.4). Aliquots containing 30 µg of protein were incubated with 3 µM GDP and 0.05 nM [^35^S]GTPγS (Specific activity –43.3 TBq/mmol, PerkinElmer USA) in the presence or absence of either SS-14, octreotide, pasireotide or somatoprim in concentrations ranging from 10^−12^ to 10^−6^ M. Assays were carried out in a final volume of 1 ml for 30 min at 30°C under continuous agitation. Nonspecific binding was determined in the presence of 10 µM unlabeled GTPγS. The incubation was terminated by the addtion of ice-cold buffer and rapid vacuum filtration through glass fiber filters as described above. Filters were rinsed twice with washing buffer (50 mM Tris-HCl, pH 7.4) and dried. A scintillation mixture was added, and radioactivity was determined unsing a β-counter (1600 TR, Packard, USA) [15].

## Results

We have recently shown that pasireotide exhibits partial agonistic properties at the sst_2_ receptor. While binding with high affinity it triggers only a partial internalization of the human sst_2_ receptor. To test the possibility that this behavior would be a general property of all multireceptor somatostatin analogs, we evaluated the internalization profile of somatoprim in comparison to pasireotide and octreotide. First, we examined human sst_2_, sst_3_ and sst_5_ receptors expressed in HEK293 cells by confocal microscopy revealing that in the absence of agonist all three somatostatin receptor subtypes were almost exclusively confined to the plasma membrane (Figure 1, *left panel*). As shown in Figure 1 (*upper panel*), octreotide and somatoprim were able to stimulate a robust endocytosis of human sst_2_ receptors similar to that seen after SS-14 exposure. In contrast, a saturating concentration of pasireotide induced only a very limited internalization of sst_2_ receptors. Conversely, examination of sst_3_-expressing cells revealed that pasireotide promoted a more pronounced receptor sequestration than octreotide, whereas somatoprim failed to stimulate any detectable sst_3_ internalization (Figure 1, *middle panel*). When sst_5_-expressing cells were exposed to SS-14, octreotide, pasireotide or somatoprim only the endogenous ligand SS-14 was able to stimulate a clearly detectable receptor endocytosis (Figure 1, *lower panel*).

Recently, we have generated a set of three phosphosite-specific antibodies, which allowed us to detect selectively the S341/S343-, the T353/T354- and the T356/T359-phosphorylated forms of the sst_2_ receptor [16], . When HEK293 cells stably expressing the human sst_2_ receptor were exposed for 5 min to SS-14, octreotide, pasireotide or somatoprim in concentrations ranging from 10^−12^ to 10^−5^ M, SS-14, octreotide and somatoprim were able to promote a robust dose-dependent phosphorylation of all three sites (Figure 2). In contrast, pasireotide stimulated only at saturating concentration a detectable phosphorylation of S341/S343 and T356/T359 but not of T353/T354. Considering the high binding affinity of pasireotide this result was unexpected and indicates that, in contrast to octreotide and somatoprim, pasireotide is a partial agonist at the human sst_2_ receptor. It also shows that the sst_2_ receptor can exist in distinct active conformations, which favor different patterns of GRK-mediated phosphorylation. Thus, considerable differences may exist between the binding and efficacy profiles of pan-somatostatin analogs. It would therefore be desirable to know the patterns of phosphorylation induced by multireceptor ligands at the level of individual somatostatin receptors. However, at present phosphosite-specific antibodies are only available for the sst_2_ receptor.

We therefore elucidated whether the carboxyl-terminal tail of the sst_2_ receptor can be used as probe to sense the activation of other somatostatin receptors. Consequently, we transplanted the carboxyl-terminal phosphorylation motif of the sst_2_ receptor to other clinically-relevant somatostatin receptors and assessed their patterns of activation using our set of three phosphosite-specific antibodies. Examination of cells expressing a sst_5_-sst_2_CT chimeric receptor revealed that SS-14 stimulated the most pronounced phosphorylation of all three sites (Figure 3 A). Pasireotide and somatoprim also promoted a robust phosphorylation of S341/S343 and T356/T359 (Figure 3 A). In contrast, octreotide induced only at saturating concentration a detectable phosphorylation of S341/S343 and T356/T359 (Figure 3 A). Interestingly, similar results were obtained at the wild-type sst_5_ receptor using a recently generated phosphosite-specific antibody to T333 validating our approach to study receptor activation (Figure 3 B). Analysis of ligand binding properties of the sst_5_-sst_2_CT chimera indicated that the transfer of the sst_2_ carboxyl-terminal tail to sst_5_ did not substantially affect the affinities for SS-14, octreotide, pasireotide or somatoprim (Table 1).

We then examined the capacity of these compounds to stimulate GTPγS binding in membrane preparations from the same cells (Figure 4). Unlike that seen in sst_2_ receptor phosphorylation assays, pasireotide was able stimulate GTPγS binding to a similar degree as octreotide or somatoprim suggesting that pasireotide is a G protein-biased ligand. In contrast, octreotide stimulated GTPγS binding in both sst_5_- and sst_5_-sst_2_CT-expressing cells to a much lesser extend than pasireotide or somatoprim suggesting it is indeed a weak partial agonist at the sst_5_ receptor. Again similar results were obtained with the wild-type sst_5_ and the sst_5_-sst_2_CT receptor.

To elucidate whether our approach can be used to directly assess the activation of a wide variety of G protein-coupled receptors, we next examined a sst_3_-sst_2_CT chimera. Examination of HEK293 cells stably expressing sst_3_-sst_2_CT receptors revealed that only pasireotide but not octreotide was able to promote a robust phosphorylation of S341/S343 and T356/T359 (Figure 5). In contrast, somatoprim failed to induce any detectable phosphorylation. Pasireotide is less potent than octreotide in inducing internalization of the sst_2_ receptor but more potent than octreotide in inducing internalization of the sst_3_ receptor. Thus, the patterns of phosphorylation of the rsst_3_-sst_2_CT chimera correlates very well with pattern internalization of the wild-type sst_3_ receptor (Figure 1). Nevertheless, it should be noted that these results were obtained with rat sst_3_-sst_2_CT receptor construct. Given the recently observed species differences for the sst_2_ receptor, these results need to be reproduced with the human sst_3_ receptor.

## Discussion

The development of new drugs targeting GPCRs is primarily focused on the discovery of compounds with nanomolar and subnanomolar binding affinities. Then indirect methods mostly assessing G protein signaling are being used to determine whether a new compound is a full or partial agonist. Accumulating evidence suggests that more than one active conformation exists for many GPCRs and that many compounds selectively stimulate specific signaling pathways [20]. Thus, there is clearly a need for methods providing more direct information on receptor activation. However, structural information is only available for a few activated receptors, and none of these has been crystallized in more than one active conformation yet [21],[22]. Determination of receptor activation using biophysical methods requires insertion of bulky fluorescent proteins into the receptor which may itself affect receptor activation [23].

In the present study, we have used the phosphorylation motif of the sst_2_ receptor to probe GPCR activation. This approach was possible due to our recent success in generating a set of three phosphosite-specific antibodies for the sst_2_ receptor which allowed us to determine distinct patterns of phosphorylation induced by different agonists. Given the unique ability of GRKs to detect only active GPCRs these distinct conformations may reflect different receptor conformations. However, phosphosite-specific antibodies are notoriously difficult to generate and are only available for a few receptors. We therefore elucidated whether the carboxyl-terminal tail of the sst_2_ receptor can be used as probe to sense the activation of other somatostatin receptors. Perhaps the most convincing evidence that this might be a valid and useful approach comes from a sst_5_-sst_2_CT chimera. In fact, the results obtained with the sst_5_-sst_2_CT chimeric receptor and the wild-type sst_5_ receptor were very similar. We have also confirmed that insertion of the sst_2_ phosphorylation motif into other somatostatin receptors did not dramatically change their binding properties with regard to the compounds tested. In addition, construction of a sst_3_-sst_2_CT chimera was also successful indicating that this approach could be used to examine the activation of a wide variety of GPCRs. Our assay can be adapted to a quantitative ELISA method and thus be applied to screening of large numbers of ligands [24]. However, for other receptors it cannot be completely ruled out that transplantation of the sst_2_CT may alter receptor function. Therefore, a functional analysis of such chimeric receptors needs to be performed in each case.

Our study also yielded valuable and previously unappreciated information about the pan-somatostatin analogs currently under clinical and preclinical examination. Pasireotide exhibited potent agonistic activity at the sst_5_ receptor but only weak partial agonistic properties at the sst_2_ receptor. Consequently, pasireotide should be classified as sst_5_-preferring ligand. Octreotide is a full agonist at the sst_2_ receptor but exhibited virtually no agonistic activity at the sst_5_ receptor. Consequently, octreotide should be classified as sst_2_-preferring ligand. In contrast, somatoprim is unique in that it was a potent agonist at both sst_2_ and sst_5_ receptors. In fact, this may provide the molecular basis for the recent observation that somatoprim can inhibit GH release in cases, which did not respond to octreotide [25].

In conclusion, we describe the use of a phosphorylation probe for direct assessment of GPCR activation and demonstrate its utility in the pharmacological characterization of novel pan-somatostatin analogs.

## Supporting Information

Table S1 **Ligand binding properties of rat, human and mutant somatostatin receptors.** Ligand binding assays were carried out as described under “Materials and Methods”. The half-maximal inhibitory concentrations (IC_50_) were analyzed by nonlinear regression curve fitting using the computer program GraphPad Prism. Data are presented as the mean of three independent experiments performed in triplicate.
(DOC)
