# Supplementary material for: A Transplantable Phosphorylation Probe for Direct Assessment of G Protein-Coupled Receptor Activation
Source: PLoS One. 2012 Jun 26;7(6):e39458. doi: 10.1371/journal.pone.0039458 (PMC3383726; doi:10.1371/journal.pone.0039458)
Supplement: Table S1 — Ligand binding properties of rat, human and mutant somatostatin receptors. Ligand binding assays were carried out as described under “Materials and Methods”. The half-maximal inhibitory concentrations (IC50) were analyzed by nonlinear regression curve fitting using the computer program GraphPad Prism. Data are presented as the mean of three independent experiments performed in triplicate. (DOC) [file pone.0039458.s001.doc]

**Supplemental Table 1**

**Ligand binding properties of rat, human and mutant somatostatin receptors.**

**Ligand binding assays were carried out as described under "Materials and Methods". The half-maximal inhibitory concentrations (IC_50_) were analyzed by nonlinear regression curve fitting using the computer program GraphPad Prism. Data are presented as the mean of three independent experiments performed in triplicate.**

|  | Ligand binding affinity IC_50_ (nM) | | |
| --- | --- | --- | --- |
|  | SS-14 | Octreotide | Pasireotide |
| human sst1 | 2.1 ± 2.3 | 403 ± 121 | 10.4 ± 4.3 |
| rat sst1 | 1.3 ± 0.8 | 367 ± 89 | 9.5 ± 5.4 |
| human sst2 | 5.7 ± 1.1 | 1.4 ± 0.3 | 21.3 ± 5.7 |
| rat sst2 | 3.5 ± 2.8 | 6.6 ± 0.2 | 19.8 ± 6.4 |
| human sst3 | 5.1 ± 1.7 | 105.0 ± 31.3 | 38.7 ± 11.7 |
| rat sst3 | 5.0 ± 2.1 | 80.2 ± 15.5 | 29.5 ± 8.3 |
| rat sst3-sst2CT | 14.1 ± 2.0 | 501.1 ± 65.2 | 31.1 ± 8.1 |
| human sst5 | 15.5 ± 2.6 | 28.9 ± 4.2 | 3.6 ± 1.5 |
| rat sst5 | 2.9 ± 2.4 | 36.3 ± 9.8 | 1.9 ± 1.3 |
